# Supplementary material for: Partitioning and Spatial Distribution of Drugs in Ocular Surface Tissues
Source: Pharmaceutics. 2021 May 4;13(5):658. doi: 10.3390/pharmaceutics13050658 (PMC8147976; doi:10.3390/pharmaceutics13050658)
Supplement: Supplementary file 1 [file pharmaceutics-13-00658-s001.zip › pharmaceutics-1183338-supplementary.pdf]

pharmaceutics

MDPI

Supplementary Materials: Partitioning and Spatial Distribution of Drugs in Ocular Surface Tissues

Anusha Balla, Seppo Auriola, Angus C. Grey, Nicholas J. Demarais, Annika Valtari, Emma M. Heikkinen, Elisa Toropainen, Arto Urtti, Kati-Sisko Vellonen and Marika Ruponen

Table S1. LC-MS/MS data acquisition parameters.

Compound	Qualifier/Quantifier	Precursor Ion (*m*/*z*)	Product Ion (*m*/*z*)	Collision Energy (V)
timolol-d5	Quantifier	322	266	15
timolol	Quantifier	317	261	20
timolol	Qualifier	317	74.2	20
betaxolol-d5	Quantifier	313	121	25
nadolol	Quantifier	310	254.1	17
nadolol	Qualifier	310	201	25
betaxolol	Quantifier	308	116.1	25
betaxolol	Qualifier	308	72.1	29
atenolol-d7	Quantifier	274	145.1	28
atenolol-d7	Qualifier	274	79.2	20
metoprolol	Quantifier	268.1	116	20
metoprolol	Qualifier	268.1	74	20
atenolol	Qualifier	267	190	21
atenolol	Quantifier	267	145	29
propranolol	Qualifier	260	183	17
propranolol	Quantifier	260	116.1	17
pindolol	Qualifier	249	171.9	17
pindolol	Quantifier	249	116.1	17

The following source conditions were applied in the MS/MS analysis of samples: positive ionization mode, capillary voltage 3.5 kV, nebulizer 25 psi, gas temp 200 °C, gas flow 16 l/min, sheath gas heater 350 °C, sheath gas flow 11 L/min, fragmentor voltage 380 V, dwell time 45 ms and cell accelerator voltage 5 V.

Table S2. Experimental and computational values for the corneal and conjunctival permeability of beta blocking drugs in the rabbit tissues. The computational values were calculated for cornea as $\text{Log } P_{app,co} = -4.6823 - 0.7670(\text{logPSA}) - 0.1346(\text{HBD}) + 3.0024(\text{Halogen ratio})$. For conjunctiva, the values were calculated as $\text{Log } P_{app,cj} = 4.1594 - 0.6121(\text{logPSA}) - 0.0792(\text{HBD}) + 3.2914(\text{Halogen ratio})$.

Compound	Log $D_{7.4}$	PSA	log PSA	HBD	Halogen ratio	Log $P_{app,co}$	[1] $P_{app,co}$ cm/s	[1] Experimental $P_{app,co}$, cm/s	Log $P_{app,cj}$	[2] $P_{app,cj}$ cm/s	[2] Experimental $P_{app,cj}$, cm/s
Propranolol	0.79	41.5	1.618	2	0	−6.193	6.4×10^{-7}	1.5×10^{-6}	−5.308	4.9×10^{-6}	5.44×10^{-6}
Betaxolol	0.43	50.7	1.705	2	0	−6.259	5.5×10^{-7}	1.6×10^{-6}	−5.361	4.4×10^{-6}	7.08×10^{-6}
Pindolol	0.06	57.3	1.758	3	0	−6.435	3.7×10^{-7}	7.5×10^{-7}	−5.473	3.4×10^{-6}	5.72×10^{-6}
Metoprolol	−0.5	50.7	1.705	2	0	−6.259	5.5×10^{-7}	na	−5.361	4.4×10^{-6}	na
Nadolol	−1.43	82	1.914	4	0	−6.689	2.0×10^{-7}	1.2×10^{-7}	−5.648	2.3×10^{-6}	1.25×10^{-6}

Timolol	−1.54	108	2.033	2	0	−6.511	3.1×10^{-7}	na	−5.562	2.7×10^{-6}	na
Atenolol	−1.76	84.6	1.927	4	0	−6.699	2.0×10^{-7}	1.7×10^{-7}	−5.656	2.2×10^{-6}	1.51×10^{-6}

PSA (polar surface area); HBD (hydrogen bond donors); $P_{app,co}$ (corneal permeability); $P_{app,cj}$ (conjunctival permeability). na = not available, [1] Ramsay et al., 2018 and [2] Ramsay et al., 2017.

Table S3. MALDI-IMS ionization efficiency determination of a drug standard mixture spotted on rabbit eye tissue. Different drug standard amounts and washing protocols were performed to thoroughly assess any apparent ionization efficiency differences. Absolute ion intensities and intensities normalized to *m*/*z* 260.1645 (propranolol) for three different tissue preparations.

Tissue	Absolute Ion Intensities (arb. units)			Normalized Intensities	
	***m*/*z* 249.1598 (Pindolol)**	***m*/*z* 260.1645 (Propranolol)**	***m*/*z* 317.1650 (Timolol)**	***m*/*z* 249.1598/ *m*/*z* 260.1645**	***m*/*z* 317.1650/ *m*/*z* 260.1645**
		Tissue Preparation 1 [a]			
Aq. humor	1.31×10^{8}	8.10×10^{7}	1.18×10^{8}	1.6	1.5
Cornea	2.06×10^{7}	2.84×10^{7}	1.33×10^{7}	0.7	0.5
Off-tissue	2.47×10^{8}	2.01×10^{8}	1.01×10^{8}	1.2	0.5
		Tissue Preparation 2 [b]			
Aq. humor	4.30×10^{7}	5.60×10^{7}	1.80×10^{7}	0.8	0.3
Cornea	1.57×10^{7}	1.47×10^{7}	1.15×10^{7}	1.1	0.8
Off-tissue	2.86×10^{7}	3.34×10^{7}	1.16×10^{7}	0.9	0.3
		Tissue Preparation 3 [c]			
Aq. humor	2.35×10^{7}	4.30×10^{7}	8.42×10^{6}	0.5	0.2
Cornea	1.17×10^{7}	5.65×10^{7}	5.44×10^{6}	0.2	0.1
Off-tissue	6.28×10^{7}	8.09×10^{7}	1.56×10^{7}	0.8	0.2

Notes**.** Reported ion intensities are the result of 50 summed spectra. The drug standard mixture was composed of pindolol:propranolol:timolol (2:1:2). [a] Mounted rabbit eye sections were washed with 50 mM ammonium formate, followed by the application of the drug standard mixture for a final concentration of ~5–10 ng/mg tissue. [b] Mounted rabbit eye sections were washed with 50 mM ammonium formate, followed by the application of the drug standard mixture for a final concentration of ~0.5–1 ng/mg tissue. [c] The drug standard mixture applied to rabbit eye sections for a final concentration of ~5–10 ng/mg tissue, followed by washing with 50 mM ammonium formate.

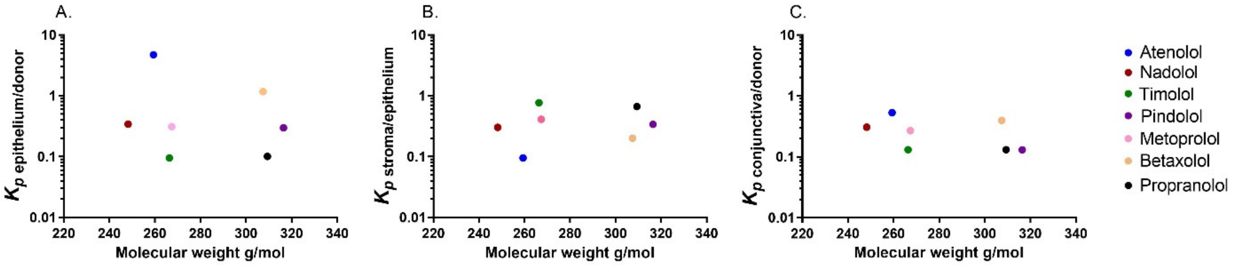

Figure S1. Relation between molecular weight of beta-blocking agents and Kp values (**A**) epithelium/donor solution, (**B**) stroma/epithelium, and (**C**) conjunctiva/donor solution.

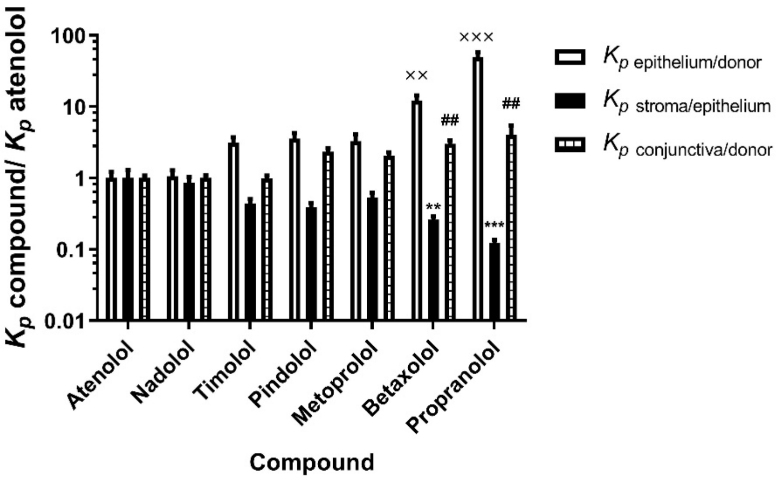

Figure S2. Estimated epithelium/donor solution, stroma/epithelium, and conjunctiva/donor solution distribution coefficients (K_P) for beta-blocking drugs normalized with atenolol after 3 h drug exposure from the epithelial side. The results are expressed as mean ± standard error of mean (SEM), *n* = 6 for cornea and *n* = 5 for conjunctiva. Statistical analysis was done by ANOVA on ranks with Dunnett's test: $^{XX}p < 0.01$, and $^{XXX}p < 0.001$ for K_P epithelium/donor solution, ** $p < 0.01$, and *** $p < 0.001$ for K_P stroma/epithelium and, $^{\#\#}p < 0.01$ for K_P conjunctiva/donor solution comparing the values of other beta-blocking agents to atenolol. Beta-blocking drugs are shown in the order from the hydrophilic compound (nadolol) to the most lipophilic one (propranolol).

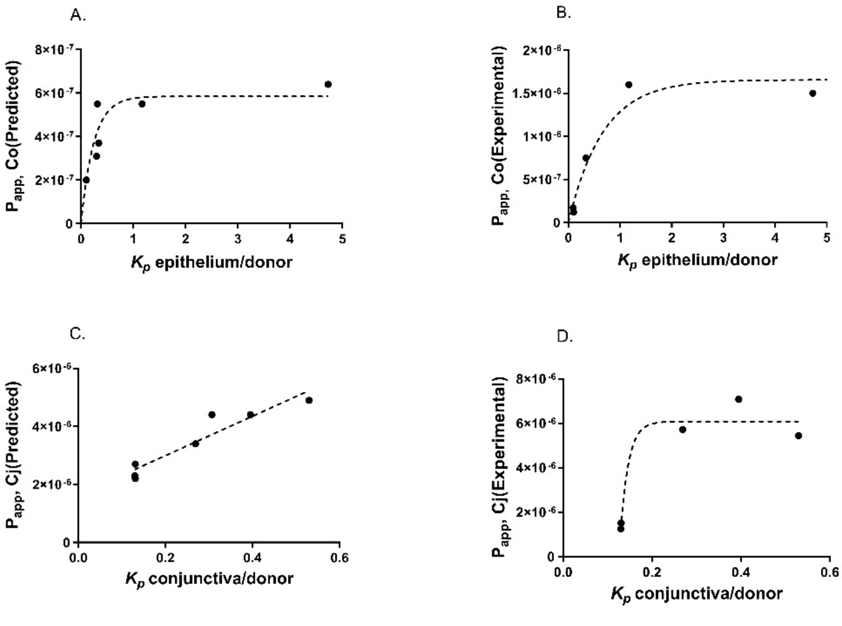

Figure S3. Relation between (**A**) predicted, (**B**) experimental corneal permeability $P_{app,co}$ and K_p epithelium/donor values and (**C**) predicted ($R^2 = 0.637$) and (**D**) experimental conjunctival permeability $P_{app,cj}$ with K_p conjunctiva/donor.

We evaluated the correlation between the drug permeability values in the cornea and conjunctiva [10,38] with the estimated K_p epithelium/donor solution and K_p conjunctiva/donor solution, respectively (Figure S2). In the case of the corneal epithelium, increasing permeability is seen at $K_p < 1$, but at $K_p > 1$ plateau is evident (Figure S 3A,B). In conjunctiva, a plateau at $K_p > 0.2$ was observed in the experimental (Figure S3D).

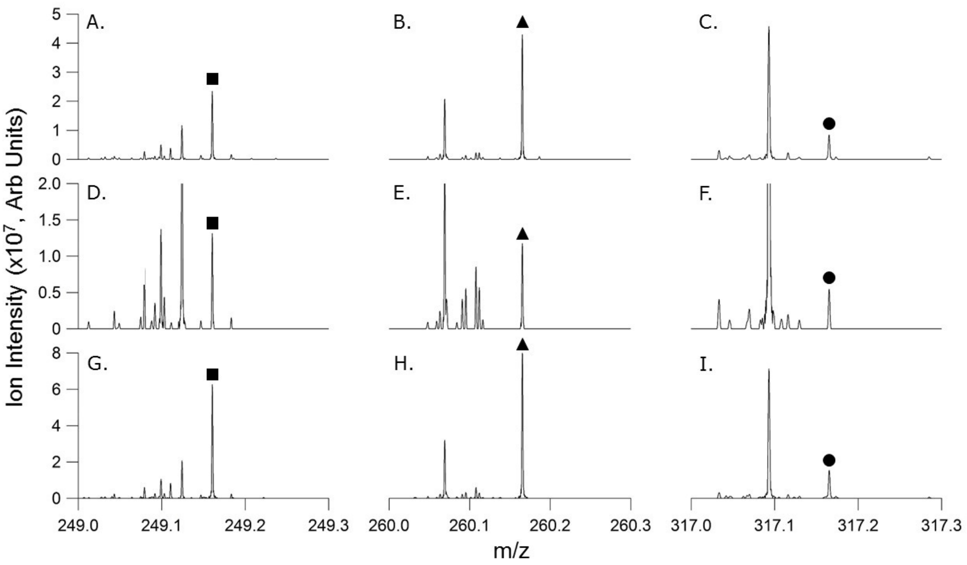

Figure S4. MALDI-IMS ionization efficiency determination of a drug standard mixture spotted on rabbit eye (**A**–**C**) aqueous humor, (**D**–**F**) cornea, and (**G**–**I**) off-tissue. Pindolol (*m/z* 249.1598, square), propranolol (*m/z* 160.1645, triangle), and timolol (*m/z* 317.1650, circle). We show these example spectra from Tissue Preparation 3 in which the drug standard was applied to rabbit eye sections for a final concentration of ~5–10 ng/mg tissue, followed by washing with 50 mM ammonium formate. The drug standard mixture was composed of pindolol:propranolol:timolol (2:1:2). Similar results were obtained from the other tissue preparation methods.
